# Social acceptance for commercialization of genetically modified food animals

**DOI:** 10.1093/nsr/nwab067

**Published:** 2021-04-21

**Authors:** Ziyao Fan, Yulian Mu, Tad Sonstegard, Xiaomei Zhai, Kui Li, Perry B Hackett, Zuoyan Zhu

**Affiliations:** Genome Analysis Laboratory of the Ministry of Agriculture and Rural Affairs, Agricultural Genomics Institute at Shenzhen, Chinese Academy of Agricultural Sciences, China; State Key Laboratory of Animal Nutrition, Institute of Animal Science, Chinese Academy of Agricultural Sciences, China; State Key Laboratory of Animal Nutrition, Institute of Animal Science, Chinese Academy of Agricultural Sciences, China; Acceligen Inc., USA; Center for Bioethics, Chinese Academy of Medical Sciences and Peking Union Medical College, China; School of Population Medicine and Public Health, Peking Union Medical College, China; Genome Analysis Laboratory of the Ministry of Agriculture and Rural Affairs, Agricultural Genomics Institute at Shenzhen, Chinese Academy of Agricultural Sciences, China; State Key Laboratory of Animal Nutrition, Institute of Animal Science, Chinese Academy of Agricultural Sciences, China; Center for Genome Engineering, Department of Genetics, Cell Biology, and Development, University of Minnesota, USA; State Key Laboratory of Freshwater Ecology and Biotechnology, Institute of Hydrobiology, Chinese Academy of Sciences, China

## Abstract

Genetically modified food animals (GMFAs) are needed to address early the cumulative effects of livestock production on the environment, and to accommodate future food demands. In 2020 China and the USA, the world's two largest economies, embarked on regulatory reforms to boost the commercialization of such animals. However, gaining social acceptance of GMFAs for commercialization remains a global challenge. We propose a framework that focuses on social license for commercialization of GMFAs by defining four classes of improvement using precision genetics: (1) animals equivalent to natural variation to obtain the improved effect of cross-breeding (ENV); (2) animals with an inactivated gene that could occur via natural mutation (ENC−); (3) animals harboring a natural genetic sequence isolated from another species (ENC+); and (4) animals with synthetic sequences encoding novel genes (BNE). Our approach can guide regulators and the public to support orderly commercialization of GMFAs.

Since late 2020, China and the United States, the world's two largest economies, have embarked on regulatory reforms to boost the regulatory development of genetically modified food animals (GMFAs). The Ministry of Agriculture and Rural Affairs of the People's Republic of China issued a circular to encourage original innovation of agriculturally important, genetically modified organisms (GMOs) as well as to standardize and regulate the transport, transfer and cross-breeding of biological materials [1]. The US Food and Drug Administration (FDA) recently approved GalSafe^TM^ pigs, the first-of-its-kind intentional genomic alteration in GM livestock for medical and/or food use [2], even though there is a proposed shift in regulatory responsibilities for GM animals from the FDA to US Department of Agriculture (USDA) under consideration [3].

Public concerns about GMFAs reflecting a lack of social acceptability have been obstructing regulatory approval of GMOs for commercialization in both countries. In China, except for papaya and cotton (cottonseed oil), no GM plant or animal has been entered into commercial cultivation and breeding for food [4]. In the United States, the lead agency does not depend on the species for plants or insects, but rather the application; the FDA is the lead agency, at the moment, for all vertebrate animals no matter the application. As in most countries, the intent of the Chinese regulatory agencies is to assure that an agricultural GM product poses no harm to either human health or the environment. However, even after a safety certificate is obtained, GMOs must obtain additional business-related certifications before commercialization can begin.

With the advent of genome editing, precise genetic changes in GMFAs can be achieved and incorporated into selective breeding programs. However, applying transformational genetics requires social license, a concept based on ethics, history, customs, etc., which are difficult to apply to scientific technologies that are evaluated in quantifiable terms. Science can define the characteristics of a GMFA but cannot answer public policy questions about whether it should be commercialized. Here, we propose a strategy to evaluate GMFAs for safety and commercialization and thereby encourage broader international support of GM agriculture.

Most industrialized countries support animal research that meets international ethical guidelines. Social evaluation for commercialization is based on the animal's attributes in terms of benefit to humans, to themselves, and to the environment, that is to help fulfill societal needs. A common social concern has been that GMFAs are unnatural, which makes them wrong. The concept of naturalistic fallacy of inferring evaluative conclusions from purely factual premises is relevant to this public perception. We propose evaluating GMFAs in terms of their natural equivalence to traditional breeding, with the understanding that GM technology can obtain a desirable genotype within a single generation. Ranking genomic alterations in GMFAs compared to present-day food animals is a method that can be intuitively understood by the public. There is precedence for this approach. In 1993, the Organization for Economic Cooperation and Development (OECD) proposed the Principle of Substantial Equivalence (SE), which states that a new food or food product should be considered equally safe to an existing food or food product if it contains essentially the same ingredients. However, this approach may defeat the whole idea of using GM technology to make desired improvements in livestock beyond those which could occur incrementally in nature. Adoption of a GM food should be evaluated by the need(s) it can safely fulfill.

Accordingly, we propose expanding the concept of SE by distinguishing four classes of GMFAs. (1) Equivalent to natural variation (ENV) achieved through GE that introduces a beneficial genetic sequence that already exists in a subpopulation of the same species to obtain the improved effect equivalent to natural crossbreeding (ENC). That is, if there are natural mutations that can mimic the changes made in ENV GMFAs, then these animals should be acceptable by a majority of the informed public. (2) ENC−, a species in which an endogenous gene is inactivated (knock-out); gene inactivation is a natural process that can be achieved efficiently using genome editing. (3) ENC+, insertion of a new genetic sequence (knock-in). ENC+ includes GMO-type changes for which the effective phenotype is predictable; currently most ENC+ GMFAs will employ site-specific insertions of the new genetic sequences to avoid questions of unknown effects from random integration. (4) Beyond Natural Equivalence (BNE), GMFAs with synthetic sequences encoding novel genes. BNE traits may derive from the introduction of novel biochemical pathways to improve the animal's productivity and/or environmental friendliness.

Given the successes of traditional breeding, obtaining social license and public acceptance for commercialization, the regulatory oversight for ENV and the ENC− GMFAs should be no greater than for conventional agricultural animals that result from cross-breeding to introduce superior traits into particular breeds otherwise adapted to specific regions or agricultural practices. Likewise, the evaluation process of ENC+ animals needs to be simplified on the basis of the existing GM regulatory system that has been in place for many years. ENC examples involving animal welfare include natural dehorning in dairy cattle by introgression of the polled sequence variation found in Angus beef cattle [5] and introduction of one of the ‘slick’ sequence variants from Criollo cattle into Holstein cows to accommodate higher temperatures found in equatorial regions [6]. An example of ENC− in sheep is an edit for inactivation of the myostatin gene (*MSTN)* that leads to both increased skeletal muscle mass and improved taste [7]. An example of beneficial ENC+ in pigs is increasing unsaturated fatty acid content by introduction of the *C. elegans Fat1* gene [8]. Addition of a CRISPR-encoding sequence to target the CP204L gene in African Swine Fever Virus (ASF) is a BNE-type genome edit because a novel, synthetic genetic sequence is introduced into the animal genome [9]. Edits of this type have enormous application potential; more than $50 billion in agricultural losses have resulted from ASF alone over the past 5 years.

In 2019, 135 million people in 55 countries experienced severe food insecurity leaving 75 million children with stunted growth and 17 million who experienced wasting. The COVID-19 pandemic is predicted to double these numbers in 2020 [10]. Social license for approval of GMFAs includes taking into consideration human loss and suffering, ecological damage and, to a lesser extent, economic consequences. Every aspect of GMFA production suggests that risks to human health and safety will be minimal. Improving efficiency of food production with GMFAs is designed to have a net positive environmental effect.

Economic considerations are vitally important to granting social license to GMFAs. Economics has always been the driving force for agricultural advancements. Sponsors of a GMFA need assurance that consumers and regulatory agencies will be receptive to the new product. The terrible specter of future food insecurity and attendant societal disruption is presently of little concern to most people in most societies. Given food shortages in coming decades, serious thinking about the commercialization of GMFAs today could ensure that BNE GMFAs will be developed by researchers and fully characterized for integration into nucleus breeding herds for when they are needed. Our collective current thinking must extend beyond the present to directly consider the future needs of everyone on our planet, which is experiencing rapidly changing climates. Staged introduction of GMFAs will be a vital contribution to future food security.
